# Increased fibrin deposition in the brains of individuals with Down syndrome and Alzheimer’s disease

**DOI:** 10.1002/alz.089882

**Published:** 2025-01-03

**Authors:** Annie Du, Lisi Flores‐Aguilar, Natalie C. Edwards, Patrick J. Lao, Jae Kyu Ryu, Katerina Akassoglou, Donna M. Wilcock, Julia Kofler, Milos D Ikonomovic, Florence Lai, Adam M. Brickman, Elizabeth Head

**Affiliations:** ^1^ University of California, Irvine, Irvine, CA USA; ^2^ Columbia University Irving Medical Center, New York, NY USA; ^3^ Columbia University, New York, NY USA; ^4^ Gladstone Institutes, UCSF, San Francisco, CA USA; ^5^ Indiana University School of Medicine, Stark Neurosciences Research Institute, Department of Neurology, Indianapolis, IN USA; ^6^ University of Pittsburgh, Pittsburgh, PA USA; ^7^ Harvard Medical School, Boston, MA USA; ^8^ The UC Irvine Institute for Memory Impairments and Neurological Disorders (UCI MIND), Irvine, CA USA

## Abstract

**Background:**

Individuals with Down syndrome (DS) have an increased genetic risk of developing Alzheimer’s disease (AD), with most adults developing AD neuropathology in their 40s. Despite having a low frequency of systemic vascular risk factors such as hypertension and atherosclerosis, adults with DS display cerebrovascular pathology, including microbleeds, microinfarcts, and cerebral amyloid angiopathy. This suggests that blood‐brain barrier (BBB) integrity may be compromised allowing the extravasation of blood proteins in the brain parenchyma. The blood coagulation factor fibrin promotes immune‐mediated neurodegeneration and is a marker of BBB disruption in a wide range of neurological diseases. This study investigated the severity of fibrin deposition as a measure of BBB integrity in the brains of adults with DS and AD pathology (DSAD). We hypothesized that fibrin deposition is increased in DSAD in comparison to neurotypical controls without DS or AD.

**Method:**

Fibrin immunoreactivity was assessed by free‐floating immunohistochemistry in 30µm tissue sections from the occipital cortex from neurotypical controls (n = 12; 41‐65 years old) and DSAD (n = 12; 46‐66 years old). Using whole slide imaging, brain sections were digitized, and the severity of fibrin deposition was scored using Aperio Imagescope.

**Result:**

Individuals with DSAD display significantly higher fibrin deposition in the white and grey matter of the occipital cortex in comparison to the age‐matched neurotypical controls (p<0.0001).

**Conclusion:**

Neurotypical controls display minimal fibrin deposition in the brain parenchyma and perivascular space. However, compared to neurotypical controls, adults with DS at advanced stages of AD neuropathology display significant fibrin deposition in the occipital cortex, suggesting that the BBB may be compromised in this population.

**Funding**: NIH U19AG068054, RF1AG079519, P30AG066519 and 23AARFD‐1022715.

